# Excess mortality among older adults institutionalized in long-term care facilities during the COVID-19 pandemic: a population-based analysis in Catalonia

**DOI:** 10.3389/fpubh.2023.1208184

**Published:** 2023-08-24

**Authors:** Laia Cases, Emili Vela, Sebastià J. Santaeugènia Gonzàlez, Joan Carles Contel, Gerard Carot-Sans, Marc Coca, Marta Pastor, Ignasi Carrasco, Conxita Barbeta, Anna Vila, Paloma Amil, Aina Plaza, Caridad Pontes, Jordi Piera-Jiménez, Jordi Amblàs

**Affiliations:** ^1^Central Catalonia Chronicity Research Group (C3RG), Centre for Health and Social Care Research (CESS), University of Vic—Central University of Catalonia (UVIC-UCC), Barcelona, Spain; ^2^Sub-Directorate General of Surveillance and Response to Public Health Emergencies, Public Health Agency of Catalonia, Generalitat of Catalonia, Barcelona, Spain; ^3^Catalan Health Service, Barcelona, Spain; ^4^Digitalization for the Sustainability of the Healthcare System (DS3), IDIBELL, Barcelona, Spain; ^5^General Directorate of Health and Research Planning, Department of Health, Generalitat de Catalunya, Barcelona, Spain; ^6^Integrated Social and Health Care Program, Generalitat de Catalunya, Barcelona, Spain; ^7^Department of Social Welfare, Generalitat de Catalunya, Barcelona, Spain; ^8^Department of Pharmacology, Therapeutics and Toxicology, Universitat Autònoma de Barcelona, Barcelona, Spain; ^9^Faculty of Informatics, Multimedia and Telecommunications, Universitat Oberta de Catalunya, Barcelona, Spain; ^10^Faculty of Medicine, University of Vic-Central University of Catalonia, Vic, Spain

**Keywords:** long-term care, COVID-19, excess mortality, older adults, nursing home

## Abstract

**Objectives:**

To assess excess mortality among older adults institutionalized in nursing homes within the successive waves of the COVID-19 pandemic in Catalonia (north-east Spain).

**Design:**

Observational, retrospective analysis of population-based central healthcare registries.

**Setting and participants:**

Individuals aged >65 years admitted in any nursing home in Catalonia between January 1, 2015, and April 1, 2022.

**Methods:**

Deaths reported during the pre-pandemic period (2015–2019) were used to build a reference model for mortality trends (a Poisson model, due to the event counting nature of the variable “mortality”), adjusted by age, sex, and clinical complexity, defined according to the adjusted morbidity groups. Excess mortality was estimated by comparing the observed and model-based expected mortality during the pandemic period (2020–2022). Besides the crude excess mortality, we estimated the standardized mortality rate (SMR) as the ratio of weekly deaths’ number observed to the expected deaths’ number over the same period.

**Results:**

The analysis included 175,497 older adults institutionalized (mean 262 days, SD 132), yielding a total of 394,134 person-years: 288,948 person-years within the reference period (2015–2019) and 105,186 within the COVID-19 period (2020–2022). Excess number of deaths in this population was 5,403 in the first wave and 1,313, 111, −182, 498, and 329 in the successive waves. The first wave on March 2020 showed the highest SMR (2.50; 95% CI 2.45–2.56). The corresponding SMR for the 2nd to 6th waves were 1.31 (1.27–1.34), 1.03 (1.00–1.07), 0.93 (0.89–0.97), 1.13 (1.10–1.17), and 1.07 (1.04–1.09). The number of excess deaths following the first wave ranged from 1,313 (2nd wave) to −182 (4th wave). Excess mortality showed similar trends for men and women. Older adults and those with higher comorbidity burden account for higher number of deaths, albeit lower SMRs.

**Conclusion:**

Excess mortality analysis suggest a higher death toll of the COVID-19 crisis in nursing homes than in other settings. Although crude mortality rates were far higher among older adults and those at higher health risk, younger individuals showed persistently higher SMR, indicating an important death toll of the COVID-19 in these groups of people.

## Introduction

Early after the first case of the severe acute respiratory syndrome coronavirus 2 (SARS-CoV-2) in December 2019, the virus spread rapidly across the globe, leading to over 600 million cases and more than six million deaths directly attributed to COVID (1). However, excess mortality analyses, which account for both direct and indirect deaths, indicate that the total death of the global health crisis could reach nearly 15 million (2). Older adults have been the population group with higher frequency of severe illness, hospitalizations, and deaths (3). Moreover, long-term care (LTC) facilities have been one of the most affected settings by the COVID-19 pandemic and account for the highest mortality rates (4–7). These figures highlighted the need for specific COVID-19 management policies for the LTC setting (3) therefore, different institutions and societies, such as the World Health Organization (WHO), the Centers for Disease Control and Prevention (CDC), and the American Geriatrics Society, among others, published guidance stating policies to protect LTC facilities, including residents, employees, and visitors (8–11).

During the first 2 years of the COVID-19 pandemic, the epidemiological and clinical characteristics of the disease have evolved because of the emergence of new strains, the introduction of vaccination and boosters, and the improvement of public health policies for containing the spread of SARS-CoV-2 in the community, healthcare centers, and LTC facilities (8–10, 12). However, most of the reports regarding the impact of COVID-19 to the LTC setting were focused on the firsts waves of the outbreak and there is little information on how the pandemic has evolved through the successive waves in this setting (4–7, 13–15).

To date, mortality of COVID-19 in the LTC setting has been primarily assessed using absolute mortality rates or comparing them between groups. Some studies, such as Veronese et al. (13) and Ballin et al. (5), compared mortality rates in LTC facilities between residents with and without COVID-19. In another study, Rescinti et al. (6) compared the mortality of residents and staff of LTC with that of community-dwelling older adults and adults not working in LTC facilities, respectively. While these reports provide a perspective of the relative impact of COVID-19 in LTC facilities compared with other population groups, mortality analyses in this setting are challenged by the high background mortality associated with clinical complexity of individuals institutionalized in LTC facilities (16). Therefore, an accurate assessment of mortality in this setting requires excess mortality analyses that take into account historical trends. This approach has been used in some studies, although most of them were constrained to the first few months of the pandemic (4, 7, 14, 17), thus losing sight of the evolving nature of the COVID-19 throughout successive waves and delayed effects of COVID-19 on mortality.

In this population-based, retrospective analysis, we have analyzed excess mortality in all nursing homes in Catalonia (north-east Spain) throughout the successive waves that occurred in the first 2 years of the pandemic.

## Methods

### Study setting and data sources

This was a retrospective analysis of administrative healthcare records of older adults institutionalized in any of the nursing homes in Catalonia (north-east Spain) between January 1, 2015, and April 1, 2022. The pre-pandemic period (years 2015–2019) was used as a reference for mortality trends to estimate the excess mortality during the pandemic period (years 2020–2022). In our area, nursing homes are defined as any permanent or temporary place (either privately or publicly owned) for people without sufficient degree of autonomy to perform daily activities, who need constant supervision (irrespective of their healthcare needs), or live in a social-family situation requiring the replacement of their home.

Institutionalized individuals were identified from the pharmaceutical invoicing registry (PIR). Catalonia provides universal healthcare to the entire population, with drugs being co-payed by the public healthcare insurance (i.e., the Catalan Health Service). For expenditure control purposes, drug dispensations to individuals institutionalized in any type of LTC facility are tagged with a specific code in the PIR. For this analysis, we screened the PIR for individuals with the specific tag for nursing homes at any time within the investigated period. For homogeneity in the population analysis of residents in nursing homes, we excluded individuals younger than 65 years because they typically correspond to groups with severe disabilities and mental health conditions. Deaths were retrieved from the central insurance registry (RCA for the Catalan *Registre Central d*’*Assegurats*). The PIR and RCA registries are linked through a unique identification number for public insurance purposes.

The study protocol was approved by the Research Ethics Committee of the University of Vic—entral University of Catalonia, which waived the obtention of individual informed consent. All data used in this analysis were handled according to the General Data Protection Regulation 2016/679 on data protection and privacy for all individuals within the European Union and the local regulatory framework regarding data protection.

### Outcomes and variables

The primary outcome of the analysis was death by any cause while being institutionalized in a nursing home during the investigated period. Other variables included age, sex, and the comorbidity burden, summarized using the adjusted morbidity groups (AMG) case-mix tool. The AMG is a population-based risk stratification tool designed to stratify the general population according to a weighted count of all chronic and recent acute diagnoses present at a given time from all conditions listed in the International Classification of Diseases (version 10 clinical modification, ICD-10-CM) (18). The tool retrieves a single index that can be used for adjusting multivariate models and stratifying the population into mutually-exclusive risk groups. Groups are build based on the index distribution across the entire population as follows: baseline risk (healthy stage, including AMG scores up to the 50th percentile of the total population), low risk (50th to 80th percentiles), moderate risk (80th to 95th percentiles), high risk (95th to 99th percentiles), and very-high risk (99th percentile and above). The AMG has shown high prediction capacity of key health outcomes, including but not limited to death, non-scheduled hospital admissions, and visits to the emergency room (19, 20). Information on comorbidities used to estimate the AMG index was retrieved from the Catalan Health Survey system, which centralizes and stores information collected from all primary care visits and hospitalizations covered by the Catalan Health Service. This service provides universal healthcare to the entire population of Catalonia. Since the Catalan Health Surveillance System was designed for invoicing purposes, the registry undergoes regular audits for data quality. Patients in this registry are identified with the same number than in the PIR and RCA registries.

### Analysis

We built an analysis dataset of person-days by considering the first and last dispensation for a given individual tagged as nursing home within the investigated period. The crude weekly mortality rate was estimated using the average number of individuals institutionalized within the given week as the denominator, and the number of deaths among this group of people as the numerator.

To account for seasonality, the expected mortality rate was computed by a building a Poisson regression analysis of weekly mortality between the 2015–2019 period, adjusted by age, sex, and comorbidity burden, summarized using the AMG risk categories. The Poisson model was considered the most appropriate because the primary variable was a count of events within a given time interval. The resulting coefficients of the regression were applied to the characteristics of individuals institutionalized within the COVID-19 period to obtain the expected mortality rate. To verify the model for expected deaths, we first plotted the expected and observed deaths for the 2015–2019 period. The excess mortality during the COVID-19 period was plotted and quantified by the difference between the observed and expected (central estimate, according to the model) number of deaths. We also estimated the standardized mortality rate (SMR) as the ratio of weekly number of deaths observed to the number of the expected deaths over the same period. In addition to the weekly excess mortality, we quantified it for each wave of the Catalan outbreak. The time intervals corresponding to each wave were defined based on the announcements of public health authorities in Catalonia. All analyses were conducted using R (21).

## Results

### Study population

**Figure 1:**
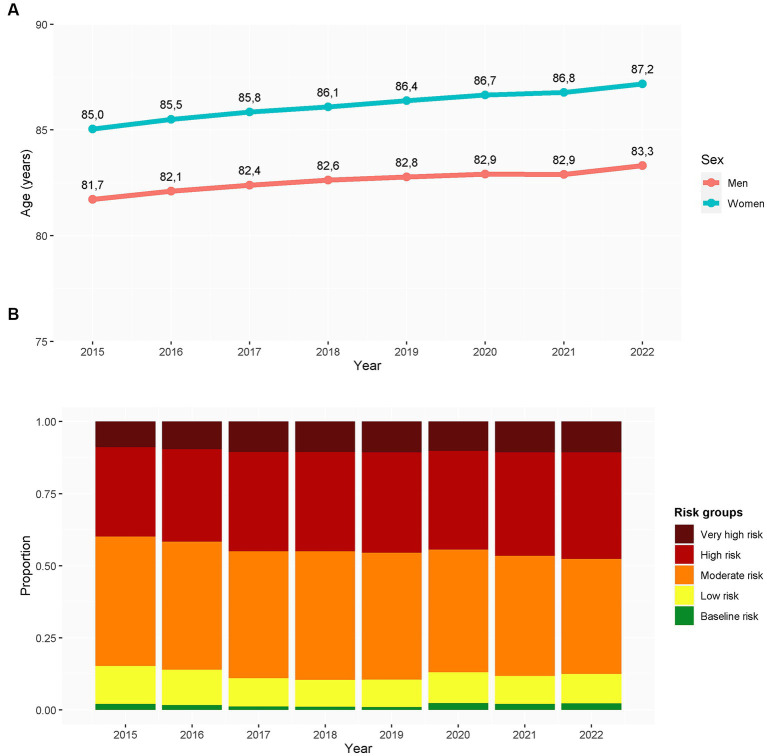
Evolution of age **(A)** and clinical complexity **(B)** of individuals older than 65 years institutionalized in nursing homes within the investigated period. The clinical complexity was assessed using the adjusted morbidity groups. Results are presented in person-years (*N* = 492,538 person-years). Clinical complexity was measured based on the adjusted morbidity groups (18, 19).

Our analysis included 175,497 persons aged 65 or older institutionalized in a nursing home at some time point between January 01, 2015, and April 1, 2022. Participants were institutionalized for a yearly mean of 262 days (SD 132), yielding a total of 394,134 person-years: 288,948 person-years within the reference period (2015–2019) and 105,186 within the COVID-19 period (2020–2022). Adults younger than 65 years accounted for 61,512 person-years (11% of the initial registry, before selecting the analysis population of older adults). Overall, the number of individuals aged 65 years or older institutionalized in nursing homes in our area showed a decreasing trend throughout the entire period (Supplementary Figure S1 and Supplementary Appendix). Table 1 summarizes the main characteristics of the study population within the reference and COVID-19 periods. The corresponding values for each year are provided in Supplementary Table S1. Individuals institutionalized in a nursing home in our area were progressively older and more complex (i.e., higher comorbidity burden, based on the AMG strata) throughout the investigated period (Figure 1). The age and sex distribution within the two periods is shown in Supplementary Figure S2.

**Table 1:** Characteristics of individuals aged >65 years institutionalized in a nursing home within the investigated period.

	Overall	2015–2019	2020–2021	*p*
Yearly stay (days), mean (SD)	262 (132)	283 (124)	219 (138)	
Person-years	394,134	288,948	105,186	
Age (years), mean (SD)	85.1 (7.46)	84.9 (7.43)	85.8 (7.49)	<0.001
Age groups, *n* (%)				<0.001
65–69	15,338 (3.9)	11,830 (4.1)	3,508 (3.3)	
70–74	25,644 (6.5)	19,304 (6.7)	6,341 (6)	
75–79	41,274 (10.5)	30,483 (10.5)	10,790 (10.3)	
80–84	80,636 (20.5)	62,459 (21.6)	18,177 (17.3)	
85–89	113,621 (28.8)	83,002 (28.7)	30,619 (29.1)	
90–94	85,955 (21.8)	60,657 (21)	25,298 (24.1)	
>94	31,666 (8)	21,213 (7.3)	10,453 (9.9)	
Sex, *n* (%)				<0.001
Men	102,050 (25.9)	75,722 (26.2)	26,328 (25)	
Women	292,084 (74.1)	213,226 (73.8)	78,858 (75)	
Risk group of clinical complexity^a^, *n* (%)				<0.001
Baseline	6,495 (1.6)	4,121 (1.4)	2,374 (2.3)	
Low	42,164 (10.7)	31,430 (10.9)	10,734 (10.2)	
Moderate	172,125 (43.7)	128,094 (44.3)	44,030 (41.9)	
High	133,340 (33.8)	96,275 (33.3)	37,065 (35.2)	
Very high	40,010 (10.2)	29,027 (10)	10,983 (10.4)	
Mortality (% person-years)	97,421 (24.7)	64,723 (22.4)	32,698 (31.1)	<0.001

Frequencies of categorical variables correspond to person-years.

a Based on the adjusted morbidity groups (18, 19).

### Excess mortality

**Figure 2:**
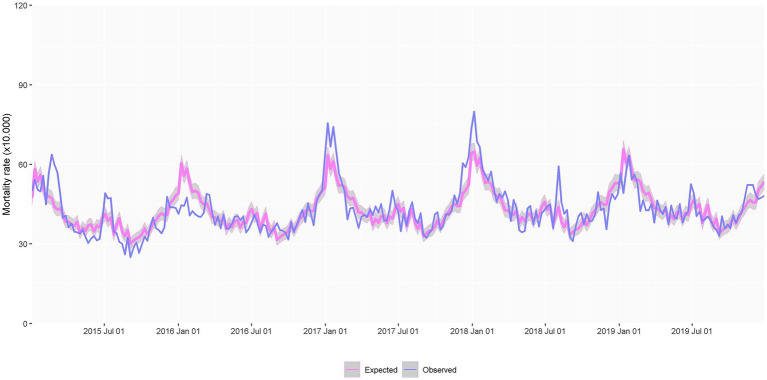
Observed and expected weekly death rate throughout the 2015–2019 period, used for developing the Poisson model of expected mortality, adjusted by sex, age, and clinical complexity (based on the adjusted morbidity groups) and accounting for seasonality (*N* = 369,016 person-years).

**Figure 3:**
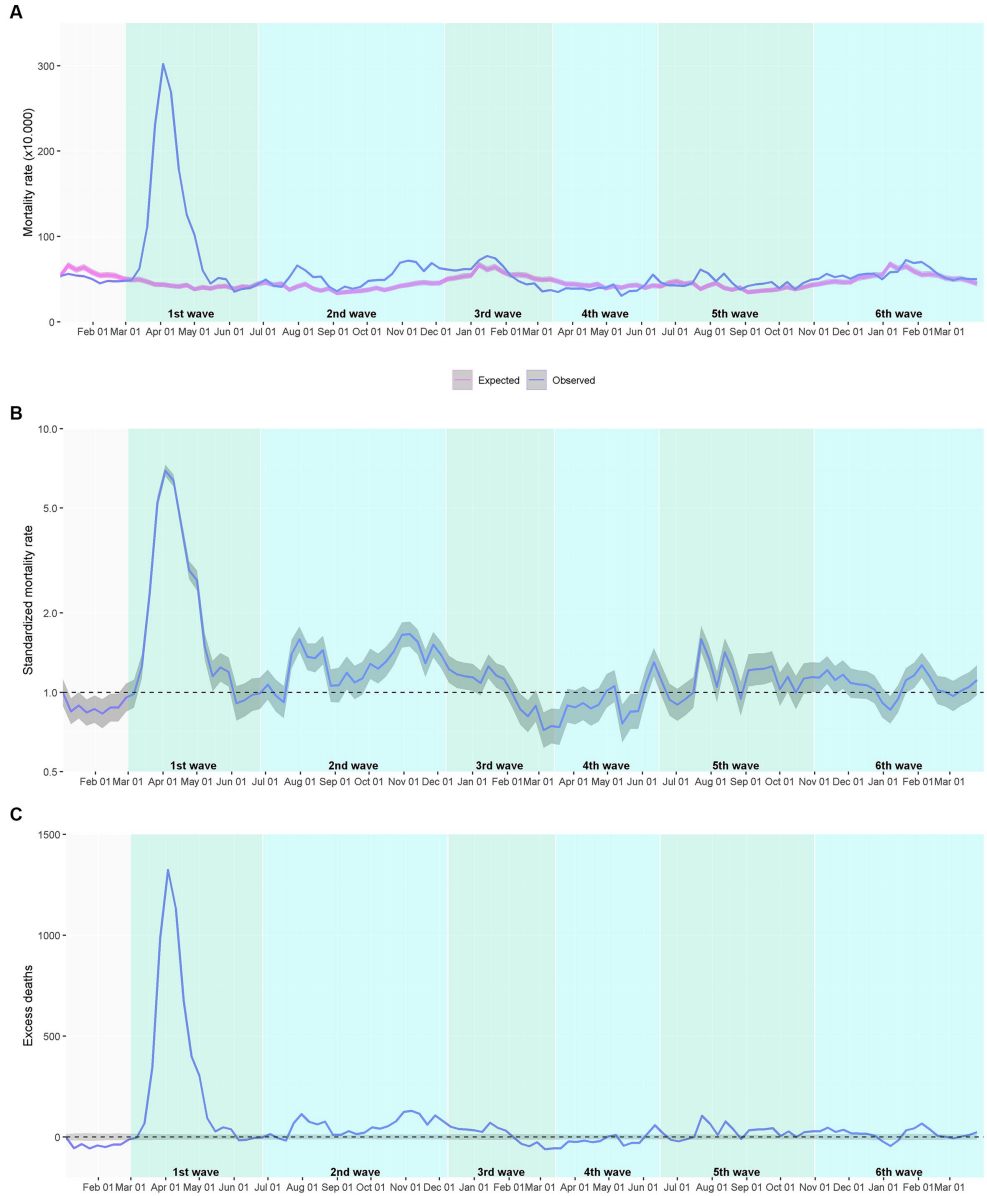
Mortality among individuals aged ≥65 years institutionalized in a nursing home during the COVID-19 outbreak. **(A)** Expected and observed weekly mortality rate. **(B)** Standardized mortality rate (blue line) with the 95% confidence interval (grey area); the dotted line shows the neutrality. **(C)** Estimated weekly excess deaths; the dotted line shows the zero excess threshold. The analysis of the investigated period (2020–2021) corresponds to 123,522 person-years.

The Poisson regression model of expected deaths generally overlapped the observed death rate within the pre-COVID-19 period (Figure 2). The model revealed a seasonal pattern, with higher rates during the winter periods and—less pervasive—the summer period. Figure 3A illustrates the expected and observed mortality rates for the overall analysis population within the COVID-19 period. The corresponding estimate of SMR and excess deaths are shown in Figures 3B,C, respectively. Supplementary Table S2 shows the average daily rates, excess deaths, and SMR in each wave. The highest mortality rate and SMR were observed during the first wave of the COVID-19 outbreak in our area. The observed mortality exceeded the expected in all waves, except the 4th one, with an onset early after the start of the vaccination campaign in nursing homes.

The excess mortality analysis according to sex showed a similar trend for men and women (Supplementary Figure S3), although the SMR was slightly higher in men for all waves (Supplementary Table S3). Regarding age, older adults accounted for higher weekly mortality rates; however, the SMR tended to be higher in younger age groups (Supplementary Figure S4). This trend was confirmed in all waves separately, although differences were more extreme in the first wave, in which SMR was 3.596 (95% CI 3.028–4.271) for the 65–69 years age group and 2.378 (2.263–2.498) for the >94 years group (Supplementary Table S4). A similar phenomenon was observed for health risk, assessed using the AMG stratification tool: the highest mortality rates were observed in the high-and very high-risk groups; however, the SMR was overall higher in the baseline and low-risk groups (Supplementary Figure S5). The corresponding analysis according to waves revealed a remarkably higher SMR among baseline risk individuals compared with very high-risk ones during the first wave: 3.822 (3.303–4.423) vs. 2.02 (1.914–2.131) (Supplementary Table S6). These differences were less pervasive in subsequent waves.

## Discussion

Our retrospective analysis of mortality in nursing homes before and during the successive waves of the COVID-19 showed a persistent excess mortality in this setting during the entire investigated period. However, important differences were observed between waves, with the first wave remarkably outstanding over the subsequent ones. The different analytical approaches reported in the literature hamper direct comparison of excess mortality values. However, in line with other studies in LTC facilities (14, 15, 17), we found that excess mortality rates in this setting are generally higher than those observed in the overall population for the European area (2). Although we could not analyze the cause of death, the limited follow-up capacity due to staff overburden in this setting during an outbreak is likely to increase also non-COVID-19 mortality, particularly associated with cardiovascular diseases (22, 23).

It is noteworthy that long-term care facilities are heterogeneous services that may differ between countries. If these differences also result in different profiles of residents (in terms of age, multimorbidity or disability), it is expected that they influence in a different way the mortality risk in the advent of a COVID-19 wave. In a population-based analysis in Catalonia, institutionalized older adults were older than the non-institutionalized counterpart (i.e., older than 65 years among the general population) (24). Although no exhaustive comparisons of LTC populations have been conducted across Europe or globally, a study comparing the characteristics of individuals institutionalized in LTC facilities in Catalonia and the UK showed similar age and similar levels of multimorbidity, dependency, and cognitive impairment between the two countries (25).

Importantly, mortality rates and excess mortality dropped in waves following the first one—but preceding the start of vaccination campaigns—, suggesting better knowledge and management of COVID-19. Although a mortality bias in this setting after the first wave cannot be ruled out, the remarkable decrease in excess mortality before vaccination suggest that containment measures implemented specifically in nursing homes at the end of the first wave (e.g., compartmentalization of affected areas, deployment of nurse case management team for enhancing integration with hospitals and intermediate care, inventory of nursing homes with limited resources for dealing with emerging outbreaks, among others) played an important role in mortality prevention. Likewise, general public health measures implemented at the community level (e.g., social distancing, contact-tracing, mask wearing) likely reduced the risk of transmission from nursing home workers to residents. The decline in excess mortality was more pervasive in the 3rd wave, matching the start of vaccination campaigns, which prioritized individuals admitted to long-term care facilities.

An exception to the positive excess mortality observed throughout the investigated period is the negative excess and SMR lower than 1 observed between February and July 2021. Although our analysis does not allow establishing causal relationships, it is of note that the vaccination campaign started in January 2021, giving priority to individuals institutionalized in nursing homes. Therefore, the period of negative excess mortality overlapped the first 6 months following vaccination in this setting. Excess mortality returned to positive values after this period, which seems consistent with the general recommendations of prioritizing the second booster vaccine in individuals who received the previous one more than 6 months ago (26). However, this negative excess mortality could also be attributed to the harvesting effect (i.e., a phenomenon observed during exogenous shocks, such as heat waves or cold spells, and characterized by an early mortality of frailest individuals, leaving to a relevant proportion of strong survivors and subsequent lower mortality rates within the period following the crisis) (27).

While the mortality rate is the epidemiological measure most frequently reported, it has to be appraised carefully in studies investigating LTC facilities because the high health risk typically observed in this setting is associated *per se* with a higher mortality rate than the general population. In this regard, we considered the standardized mortality rate (SMR) a more valuable measure to understand mortality observed during the COVID-19 pandemic relative to the historical trend for the same population group. This analysis revealed that while crude mortality rates were higher among older adults and those at higher health risk, younger and lower risk groups tended to higher SMR, particularly in the first wave. This finding suggest that in relative terms, the death toll was higher among groups with overall lower health risk, for which lower mortality would have been expected without a pandemic context. This phenomenon was also observed when assessing the effect of frailty in COVID-19 prognosis among hospitalized older adults (28).

Our analysis was strengthened by the population-based approach. Thanks to the integrated and centralized management of drugs, co-payed by the public health insurer, we could identify all individuals living in nursing homes (either private or public) in our country and link them with clinical and basic sociodemographic information. This advantage underscores the importance of data collection and interoperability, which if available in real time, could help monitoring of centers. However, our study has some limitations that should be taken into account when interpreting the results. First, we could stratify the population according to their clinical and demographic characteristics but not according to the type of nursing home, which may have also played a role in the observed mortality trends. Future studies including this perspective are warranted. Second, although we provide comparative information between waves, our analysis was not intended to understand the reasons behind these differences. Hence, although specific containment measures for nursing homes might have played a role, other factors relevant for explaining mortality, such as local COVID-19 incidence or the timings in the introduction of vaccines and boosters, may have also contributed to these differences (29). Finally, it is worth mentioning that the concept of nursing home may vary between countries. In our area, the lack of social/family support is an important driver for institutionalization (often with higher influence than the clinical condition); thus, excess mortality figures may differ in nursing homes primarily used for healthcare delivery.

In summary, the excess mortality and standardized mortality rate provide an accurate view of mortality associated with COVID-19 in the LTC setting, which takes into account the mortality trends typically high in this setting. Our analysis showed that mortality observed in the first wave of the COVID-19 clearly outstood over subsequent waves, although excess mortality was observed throughout the investigated period. Although crude mortality rates were far higher among older adults and those at higher health risk, younger individuals showed persistently higher SMR, suggesting an important death toll of the COVID-19 in these groups of people. This finding encourages comprehensive shielding plans that take into account groups at different risk levels. Our report provides an accurate quantification of excess mortality in nursing homes during the COVID-19 and encourages using relative measures of mortality for assessments in this setting.

## Data availability statement

The original contributions presented in the study are included in the article/Supplementary material, further inquiries can be directed to the corresponding author.

## Ethics statement

The studies involving humans were approved by Research Ethics Committee of the University of Vic—Central University of Catalonia (Spain). The studies were conducted in accordance with the local legislation and institutional requirements. Written informed consent for participation was not required from the participants or the participants’ legal guardians/next of kin in accordance with the national legislation and institutional requirements.

## Author contributions

LC, EV, SS, JC, GC-S, CP, and JA contributed to the study design and conception. LC, EV, MC, and MP contributed to data curation and analysis. LC, EV, SS, JC, GC-S, MC, MP, IC, CB, AV, PA, AP, CP, JP-J, and JA contributed to interpretation of the data and results and critically revised the manuscript draft for important intellectual content. LC, EV, GC-S, and JA contributed to the first manuscript draft. All authors contributed to the article and approved the submitted version.

## Conflict of interest

The authors declare that the research was conducted in the absence of any commercial or financial relationships that could be construed as a potential conflict of interest.

## Publisher’s note

All claims expressed in this article are solely those of the authors and do not necessarily represent those of their affiliated organizations, or those of the publisher, the editors and the reviewers. Any product that may be evaluated in this article, or claim that may be made by its manufacturer, is not guaranteed or endorsed by the publisher.
