# Supplementary material for: Excess mortality among older adults institutionalized in long-term care facilities during the COVID-19 pandemic: a population-based analysis in Catalonia
Source: Front Public Health. 2023 Aug 24;11:1208184. doi: 10.3389/fpubh.2023.1208184 (PMC10507684; doi:10.3389/fpubh.2023.1208184)

**Excess mortality in older people institutionalized in long-term care facilities during two years of the COVID-19 pandemic: a retrospective analysis of all nursing homes in a 7.5-million population**

Supplementary appendix

Contents

[**Table S1**. Characteristics of individuals aged >65 years institutionalized in a nursing home within the investigated period (years) 2](#_Toc127866917)

[**Table S2**. Excess mortality analysis according to wave 4](#_Toc127866918)

[**Table S3**. Excess mortality analysis according to wave and gender 5](#_Toc127866919)

[**Table S4**. Excess mortality analysis according to wave and age 6](#_Toc127866920)

[**Table S5**. Excess mortality analysis according to wave and health risk 8](#_Toc127866921)

[**Figure S1**. Progression of the person-years of individuals aged 65 years or older institutionalized in a nursing home. The count for 2022 ends on April. 9](#_Toc127866922)

[**Figure S2**. Age and sex distribution of the population within the two periods. 10](#_Toc127866923)

[**Figure S3.** Mortality among individuals aged ≥65 years institutionalized in a nursing home during the COVID-19 outbreak, according to sex. 11](#_Toc127866924)

[**Figure S4.** Mortality among individuals aged ≥65 years institutionalized in a nursing home during the COVID-19 outbreak, according to age groups. 12](#_Toc127866925)

[**Figure S5.** Mortality among individuals aged ≥65 years institutionalized in a nursing home during the COVID-19 outbreak, according to the health risk based on the adjusted morbidity groups. 15](#_Toc127866926)

## **Table S1**. Characteristics of individuals aged >65 years institutionalized in a nursing home within the investigated period (years)

|  | **Total** | **2015** | **2016** | **2017** | **2018** | **2019** | **2020** | **2021** | **2022*** | ***P*** |
| --- | --- | --- | --- | --- | --- | --- | --- | --- | --- | --- |
| Yearly stay (days), mean (SD) | 262 (132) | 276 (129) | 285 (125) | 283 (124) | 284 (123) | 288 (119) | 272 (128) | 274 (125) | 82.3 (20.2) |  |
| Person-year | 394134 | 59088 | 58908 | 58100 | 56970 | 55882 | 48437 | 45513 | 11237 |  |
| Age (years), mean (SD) | 85.1 (7.46) | 84.2 (7.47) | 84.6 (7.41) | 84.9 (7.38) | 85.2 (7.38) | 85.4 (7.44) | 85.7 (7.43) | 85.8 (7.53) | 86.2 (7.60) | <0.001 |
| Age groups, n (%) |  |  |  |  |  |  |  |  |  | <0.001 |
| 65-69 | 15338 (3.9) | 2863 (4.8) | 2505 (4.3) | 2280 (3.9) | 2167 (3.8) | 2014 (3.6) | 1594 (3.3) | 1550 (3.4) | 364 (3.2) |  |
| 70-74 | 25644 (6.5) | 4362 (7.4) | 3942 (6.7) | 3782 (6.5) | 3702 (6.5) | 3517 (6.3) | 2951 (6.1) | 2741 (6) | 649 (5.8) |  |
| 75-79 | 41274 (10.5) | 7113 (12) | 6601 (11.2) | 5967 (10.3) | 5409 (9.5) | 5394 (9.7) | 5064 (10.5) | 4623 (10.2) | 1104 (9.8) |  |
| 80-84 | 80636 (20.5) | 13742 (23.3) | 13202 (22.4) | 12693 (21.8) | 11965 (21) | 10856 (19.4) | 8550 (17.7) | 7843 (17.2) | 1784 (15.9) |  |
| 85-89 | 113621 (28.8) | 16233 (27.5) | 16796 (28.5) | 16686 (28.7) | 16765 (29.4) | 16522 (29.6) | 14254 (29.4) | 13184 (29) | 3181 (28.3) |  |
| 90-94 | 85955 (21.8) | 11224 (19) | 11942 (20.3) | 12430 (21.4) | 12425 (21.8) | 12636 (22.6) | 11431 (23.6) | 11019 (24.2) | 2848 (25.3) |  |
| >94 | 31666 (8) | 3551 (6) | 3920 (6.7) | 4262 (7.3) | 4537 (8) | 4943 (8.8) | 4593 (9.5) | 4553 (10) | 1307 (11.6) |  |
| Sex, n (%) |  |  |  |  |  |  |  |  |  | <0.001 |
| Men | 102050 (25.9) | 15623 (26.4) | 15435 (26.2) | 15169 (26.1) | 14889 (26.1) | 14606 (26.1) | 12158 (25.1) | 11335 (24.9) | 2835 (25.2) |  |
| Women | 292084 (74.1) | 43465 (73.6) | 43473 (73.8) | 42931 (73.9) | 42081 (73.9) | 41276 (73.9) | 36279 (74.9) | 34178 (75.1) | 8402 (74.8) |  |
| Risk group^a^, n (%) |  |  |  |  |  |  |  |  |  | <0.001 |
| Baseline | 6495 (1.6) | 1214 (2.1) | 1012 (1.7) | 706 (1.2) | 622 (1.1) | 567 (1) | 1153 (2.4) | 964 (2.1) | 257 (2.3) |  |
| Low | 42164 (10.7) | 7784 (13.2) | 7236 (12.3) | 5712 (9.8) | 5346 (9.4) | 5351 (9.6) | 5173 (10.7) | 4411 (9.7) | 1151 (10.2) |  |
| Moderate | 172125 (43.7) | 26514 (44.9) | 26120 (44.3) | 25550 (44) | 25352 (44.5) | 24558 (43.9) | 20591 (42.5) | 18962 (41.7) | 4477 (39.8) |  |
| High | 133340 (33.8) | 18293 (31) | 18874 (32) | 20011 (34.4) | 19639 (34.5) | 19458 (34.8) | 16584 (34.2) | 16330 (35.9) | 4151 (36.9) |  |
| Very high | 40010 (10.2) | 5283 (8.9) | 5666 (9.6) | 6121 (10.5) | 6011 (10.6) | 5948 (10.6) | 4936 (10.2) | 4846 (10.6) | 1201 (10.7) |  |
| Mortality (% person-years) | 97421 (24.7) | 12168 (20.6) | 12219 (20.7) | 13839 (23.8) | 13555 (23.8) | 12942 (23.2) | 17954 (37.1) | 11365 (25) | 3379 (30.1) | <0.001 |

## **Table S2**. Excess mortality analysis according to wave

| **Wave** | **Days** | **N** | **Deaths** | **Daily rate (x10,000)** | **Excess** | **SMR (95% CI)** |
| --- | --- | --- | --- | --- | --- | --- |
| 1 | 115 | 51002 | 8997 | 15.3 | 5403 | 2.503 (2.452 - 2.556) |
| 2 | 155 | 47761 | 5611 | 7.6 | 1313 | 1.306 (1.272 - 1.340) |
| 3 | 92 | 47585 | 3573 | 8.2 | 111 | 1.032 (0.999 - 1.066) |
| 4 | 93 | 44250 | 2327 | 5.7 | -182 | 0.928 (0.891 - 0.966) |
| 5 | 134 | 48210 | 4259 | 6.6 | 498 | 1.132 (1.099 - 1.167) |
| 6 | 147 | 45900 | 5427 | 8 | 329 | 1.065 (1.037 - 1.093) |

**CI:** confidence interval. **SMR:** standardized mortality rate

## **Table S3**. Excess mortality analysis according to wave and gender

| **Gender** | **N** | **Deaths** | **Daily rate (x10,000)** | **Excess** | **SMR (95% CI)** |
| --- | --- | --- | --- | --- | --- |
| **1st wave (115 days)** | | | | | |
| Male | 12836 | 2911 | 19.7 | 1806 | 2.636 (2.541 - 2.733) |
| Female | 38166 | 6086 | 13.9 | 3597 | 2.445 (2.384 - 2.507) |
| **2nd wave (155 days)** | | | | | |
| Male | 11886 | 1742 | 9.5 | 445 | 1.343 (1.282 - 1.408) |
| Female | 35876 | 3869 | 7 | 868 | 1.289 (1.249 - 1.331) |
| **3rd wave (92 days)** | | | | | |
| Male | 11789 | 1179 | 10.9 | 141 | 1.136 (1.073 - 1.203) |
| Female | 35796 | 2394 | 7.3 | -31 | 0.987 (0.949 - 1.028) |
| **4th wave (93 days)** | | | | | |
| Male | 10994 | 751 | 7.3 | -2 | 0.997 (0.928 - 1.071) |
| Female | 33256 | 1576 | 5.1 | -179 | 0.898 (0.855 - 0.943) |
| **5th wave (134 days)** | | | | | |
| Male | 12099 | 1348 | 8.3 | 210 | 1.185 (1.123 - 1.250) |
| Female | 36112 | 2911 | 6 | 287 | 1.11 (1.070 - 1.151) |
| **6th wave (147 days)** | | | | | |
| Male | 11577 | 1740 | 10.2 | 170 | 1.109 (1.058 - 1.162) |
| Female | 34323 | 3687 | 7.3 | 158 | 1.045 (1.012 - 1.079) |

**CI:** confidence interval. **SMR:** standardized mortality rate

## **Table S4**. Excess mortality analysis according to wave and age

| **Age group** | **N** | **Deaths** | **Daily rate (x10,000)** | **Excess** | **SMR (95% CI)** |
| --- | --- | --- | --- | --- | --- |
| **1st wave (115 days)** | | | | | |
| 65-69 | 1618 | 130 | 7 | 94 | 3.596 (3.028 - 4.271) |
| 70-74 | 2893 | 268 | 8.1 | 181 | 3.086 (2.738 - 3.478) |
| 75-79 | 4977 | 614 | 10.7 | 412 | 3.046 (2.814 - 3.297) |
| 80-84 | 8591 | 1297 | 13.1 | 842 | 2.85 (2.699 - 3.009) |
| 85-89 | 14733 | 2523 | 14.9 | 1506 | 2.48 (2.385 - 2.579) |
| 90-94 | 12631 | 2584 | 17.8 | 1452 | 2.283 (2.196 - 2.372) |
| >94 | 5559 | 1581 | 24.7 | 916 | 2.378 (2.263 - 2.498) |
| **2nd wave (155 days)** | | | | | |
| 65-69 | 1540 | 91 | 3.8 | 47 | 2.064 (1.681 - 2.535) |
| 70-74 | 2715 | 188 | 4.5 | 85 | 1.827 (1.584 - 2.108) |
| 75-79 | 4560 | 375 | 5.3 | 143 | 1.616 (1.460 - 1.788) |
| 80-84 | 7977 | 793 | 6.4 | 257 | 1.478 (1.379 - 1.585) |
| 85-89 | 13713 | 1517 | 7.1 | 315 | 1.262 (1.200 - 1.327) |
| 90-94 | 11998 | 1674 | 9 | 299 | 1.217 (1.161 - 1.277) |
| >94 | 5258 | 973 | 11.9 | 168 | 1.209 (1.135 - 1.287) |
| **3rd wave (92 days)** | | | | | |
| 65-69 | 1551 | 51 | 3.6 | 14 | 1.382 (1.050 - 1.819) |
| 70-74 | 2702 | 104 | 4.2 | 20 | 1.233 (1.017 - 1.494) |
| 75-79 | 4602 | 236 | 5.6 | 48 | 1.254 (1.104 - 1.425) |
| 80-84 | 7842 | 496 | 6.9 | 69 | 1.161 (1.063 - 1.268) |
| 85-89 | 13542 | 944 | 7.6 | -18 | 0.981 (0.920 - 1.046) |
| 90-94 | 11994 | 1122 | 10.2 | 17 | 1.015 (0.957 - 1.076) |
| >94 | 5352 | 620 | 12.6 | -38 | 0.942 (0.871 - 1.019) |
| **4th wave (93 days)** | | | | | |
| 65-69 | 1441 | 34 | 2.5 | 7 | 1.264 (0.903 - 1.768) |
| 70-74 | 2525 | 72 | 3.1 | 11 | 1.172 (0.930 - 1.476) |
| 75-79 | 4260 | 168 | 4.2 | 33 | 1.243 (1.068 - 1.445) |
| 80-84 | 7168 | 329 | 4.9 | 24 | 1.079 (0.969 - 1.202) |
| 85-89 | 12541 | 601 | 5.2 | -91 | 0.869 (0.802 - 0.941) |
| 90-94 | 11213 | 692 | 6.6 | -110 | 0.863 (0.801 - 0.930) |
| >94 | 5102 | 431 | 9.1 | -56 | 0.886 (0.806 - 0.973) |
| **5th wave (134 days)** | | | | | |
| 65-69 | 1546 | 63 | 3 | 24 | 1.608 (1.256 - 2.058) |
| 70-74 | 2759 | 139 | 3.8 | 47 | 1.507 (1.276 - 1.780) |
| 75-79 | 4650 | 307 | 4.9 | 104 | 1.514 (1.354 - 1.694) |
| 80-84 | 7687 | 558 | 5.4 | 111 | 1.249 (1.150 - 1.357) |
| 85-89 | 13631 | 1167 | 6.4 | 136 | 1.132 (1.069 - 1.199) |
| 90-94 | 12284 | 1255 | 7.6 | 48 | 1.04 (0.984 - 1.099) |
| >94 | 5653 | 770 | 10.2 | 27 | 1.037 (0.966 - 1.112) |
| **6th wave (147 days)** | | | | | |
| 65-69 | 1470 | 87 | 4 | 35 | 1.678 (1.360 - 2.070) |
| 70-74 | 2616 | 166 | 4.3 | 42 | 1.344 (1.154 - 1.565) |
| 75-79 | 4451 | 380 | 5.8 | 104 | 1.376 (1.244 - 1.522) |
| 80-84 | 7170 | 654 | 6.2 | 59 | 1.099 (1.018 - 1.187) |
| 85-89 | 12909 | 1466 | 7.7 | 84 | 1.06 (1.008 - 1.116) |
| 90-94 | 11768 | 1632 | 9.4 | -9 | 0.994 (0.947 - 1.044) |
| >94 | 5515 | 1042 | 12.9 | 14 | 1.014 (0.954 - 1.077) |

**CI:** confidence interval. **SMR:** standardized mortality rate

## **Table S5**. Excess mortality analysis according to wave and health risk

| **Health risk** ^1^ | **N** | **Deaths** | **Daily rate (x10,000)** | **Excess** | **SMR (95% CI)** |
| --- | --- | --- | --- | --- | --- |
| **1st wave (115 days)** | | | | | |
| Baseline | 1194 | 180 | 13.1 | 133 | 3.822 (3.303 - 4.423) |
| Low | 5395 | 758 | 12.2 | 528 | 3.293 (3.067 - 3.536) |
| Moderate | 21598 | 3348 | 13.5 | 2107 | 2.697 (2.608 - 2.790) |
| High | 17547 | 3378 | 16.7 | 1962 | 2.386 (2.307 - 2.468) |
| Very high | 5268 | 1333 | 22 | 673 | 2.02 (1.914 - 2.131) |
| **2nd wave (155 days)** | | | | | |
| Baseline | 1165 | 82 | 4.5 | 23 | 1.385 (1.115 - 1.720) |
| Low | 5204 | 437 | 5.4 | 152 | 1.533 (1.396 - 1.683) |
| Moderate | 20493 | 2187 | 6.9 | 669 | 1.44 (1.381 - 1.502) |
| High | 16203 | 2110 | 8.4 | 430 | 1.256 (1.204 - 1.311) |
| Very high | 4697 | 795 | 10.9 | 40 | 1.053 (0.982 - 1.128) |
| **3rd wave (92 days)** | | | | | |
| Baseline | 999 | 36 | 3.9 | -5 | 0.879 (0.634 - 1.218) |
| Low | 4676 | 215 | 5 | 12 | 1.059 (0.927 - 1.211) |
| Moderate | 19852 | 1200 | 6.6 | 24 | 1.021 (0.965 - 1.080) |
| High | 17031 | 1464 | 9.3 | 62 | 1.044 (0.992 - 1.099) |
| Very high | 5027 | 658 | 14.2 | 17 | 1.027 (0.951 - 1.108) |
| **4th wave (93 days)** | | | | | |
| Baseline | 900 | 34 | 4.1 | 5 | 1.187 (0.848 - 1.661) |
| Low | 4222 | 137 | 3.5 | -6 | 0.957 (0.810 - 1.132) |
| Moderate | 18335 | 744 | 4.4 | -101 | 0.881 (0.820 - 0.947) |
| High | 15986 | 964 | 6.5 | -55 | 0.946 (0.888 - 1.007) |
| Very high | 4806 | 448 | 10 | -25 | 0.947 (0.864 - 1.039) |
| **5th wave (134 days)** | | | | | |
| Baseline | 1040 | 64 | 4.6 | 19 | 1.412 (1.105 - 1.804) |
| Low | 4710 | 273 | 4.3 | 51 | 1.232 (1.094 - 1.387) |
| Moderate | 20138 | 1524 | 5.6 | 241 | 1.188 (1.130 - 1.249) |
| High | 17222 | 1716 | 7.4 | 197 | 1.13 (1.078 - 1.185) |
| Very high | 5101 | 682 | 10 | -11 | 0.984 (0.913 - 1.061) |
| **6th wave (147 days)** | | | | | |
| Baseline | 1060 | 68 | 4.4 | 2 | 1.036 (0.817 - 1.314) |
| Low | 4667 | 386 | 5.6 | 75 | 1.242 (1.124 - 1.372) |
| Moderate | 18709 | 1786 | 6.5 | 99 | 1.059 (1.011 - 1.109) |
| High | 16639 | 2220 | 9.1 | 122 | 1.058 (1.015 - 1.103) |
| Very high | 4825 | 967 | 13.6 | 31 | 1.033 (0.970 - 1.100) |

**CI:** confidence interval. **SMR:** standardized mortality rate

^1^ Based on the adjusted morbidity groups index. Groups are build based on the index distribution across the entire population as follows: baseline risk (healthy stage, including GMA scores up to the 50th percentile of the total population), low risk (50th to 80th percentiles), moderate risk (80th to 95th percentiles), and high risk (above the 95th percentile) .

## **Figure S1**. Progression of the person-years of individuals aged 65 years or older institutionalized in a nursing home. The count for 2022 ends on April.


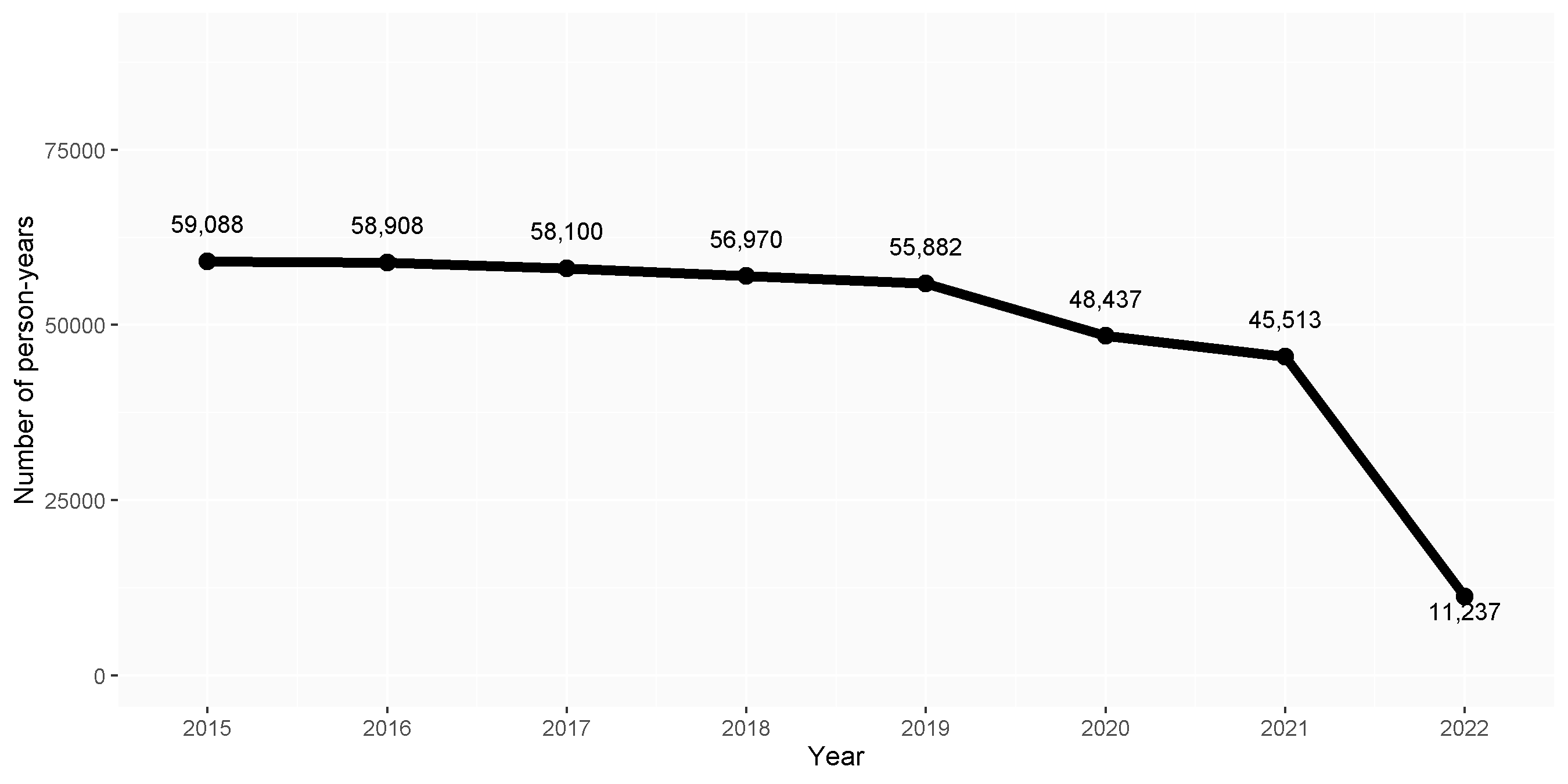


## **Figure S2**. Age and sex distribution of the population within the two periods.


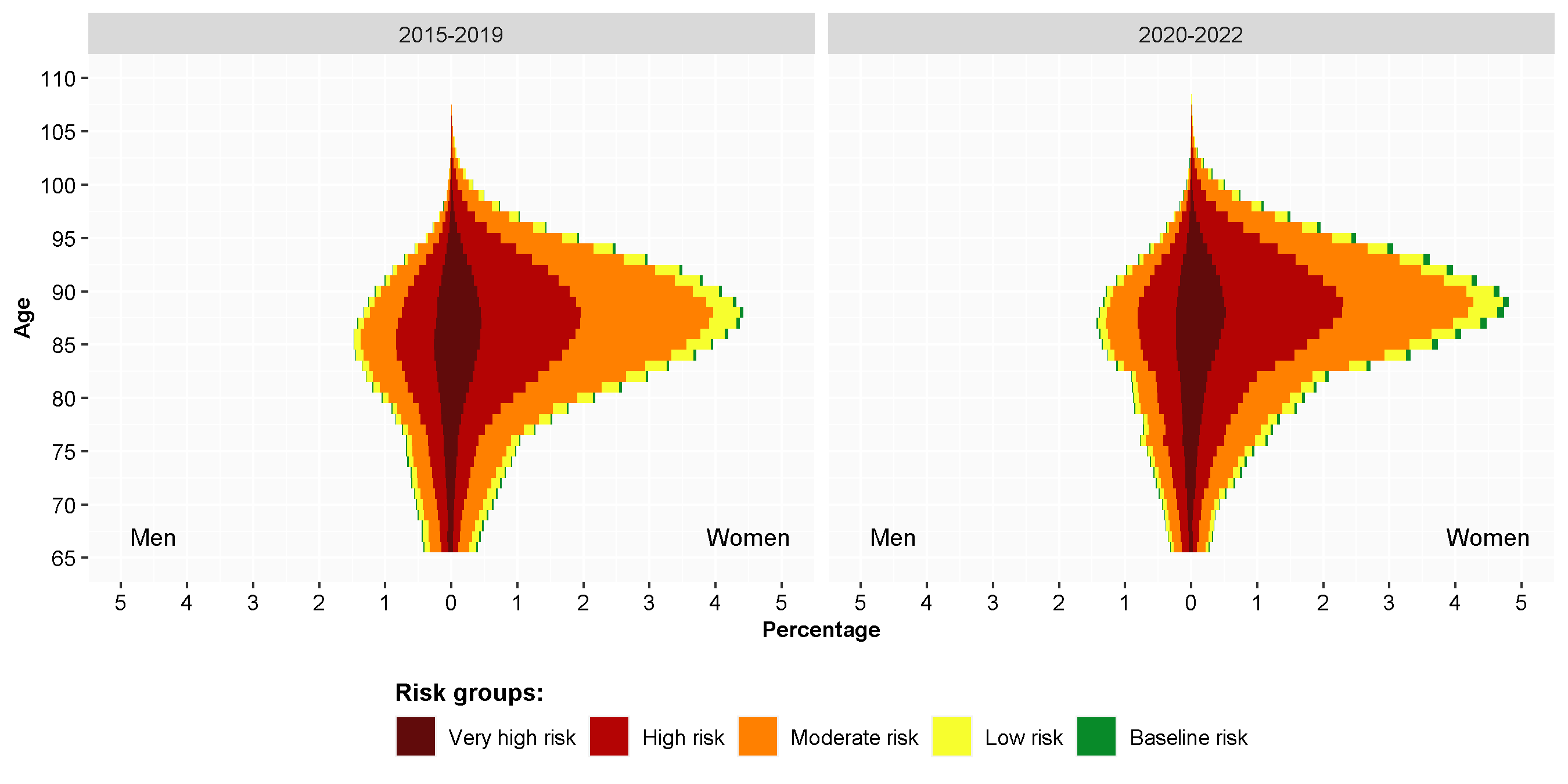


## **Figure S3.** Mortality among individuals aged ≥65 years institutionalized in a nursing home during the COVID-19 outbreak, according to sex.

**A**: Expected and observed weekly mortality rate. **B**: standardized mortality rate (blue line) with the 95% confidence interval (grey area); the dotted line shows the neutrality. **C:** estimated weekly excess deaths; the dotted line shows the zero excess threshold.

**A**


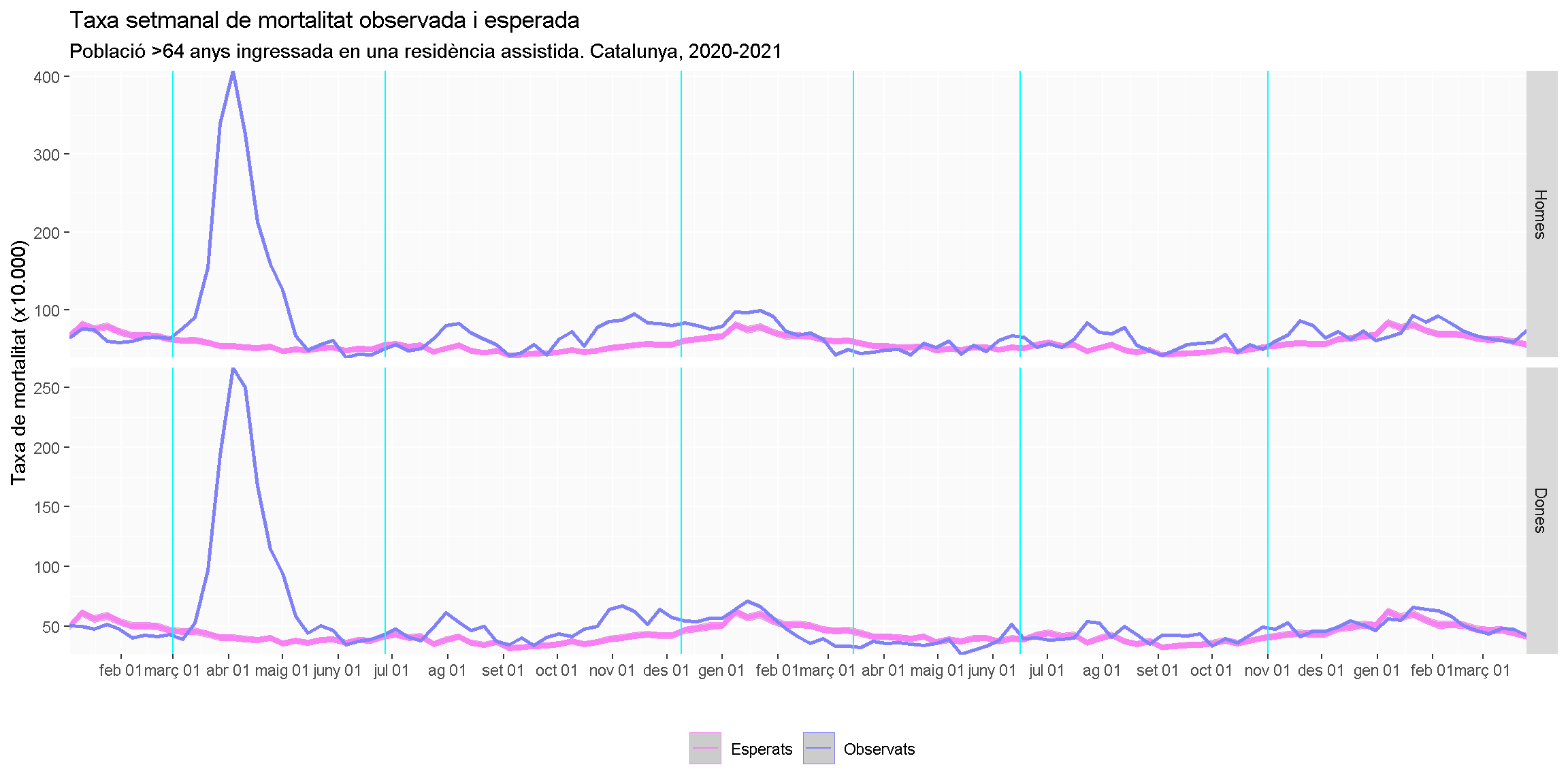


**B**


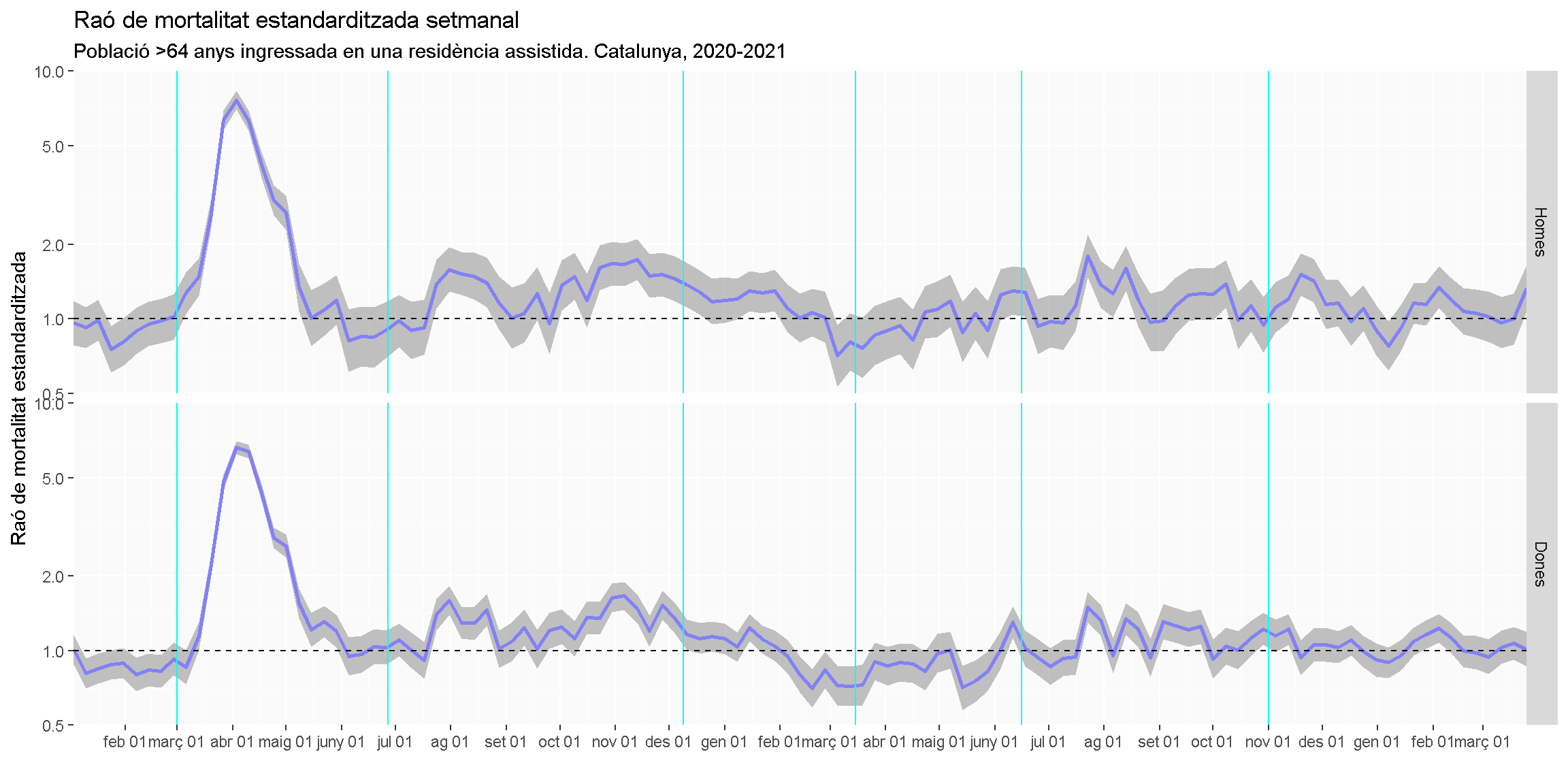


**C**

**
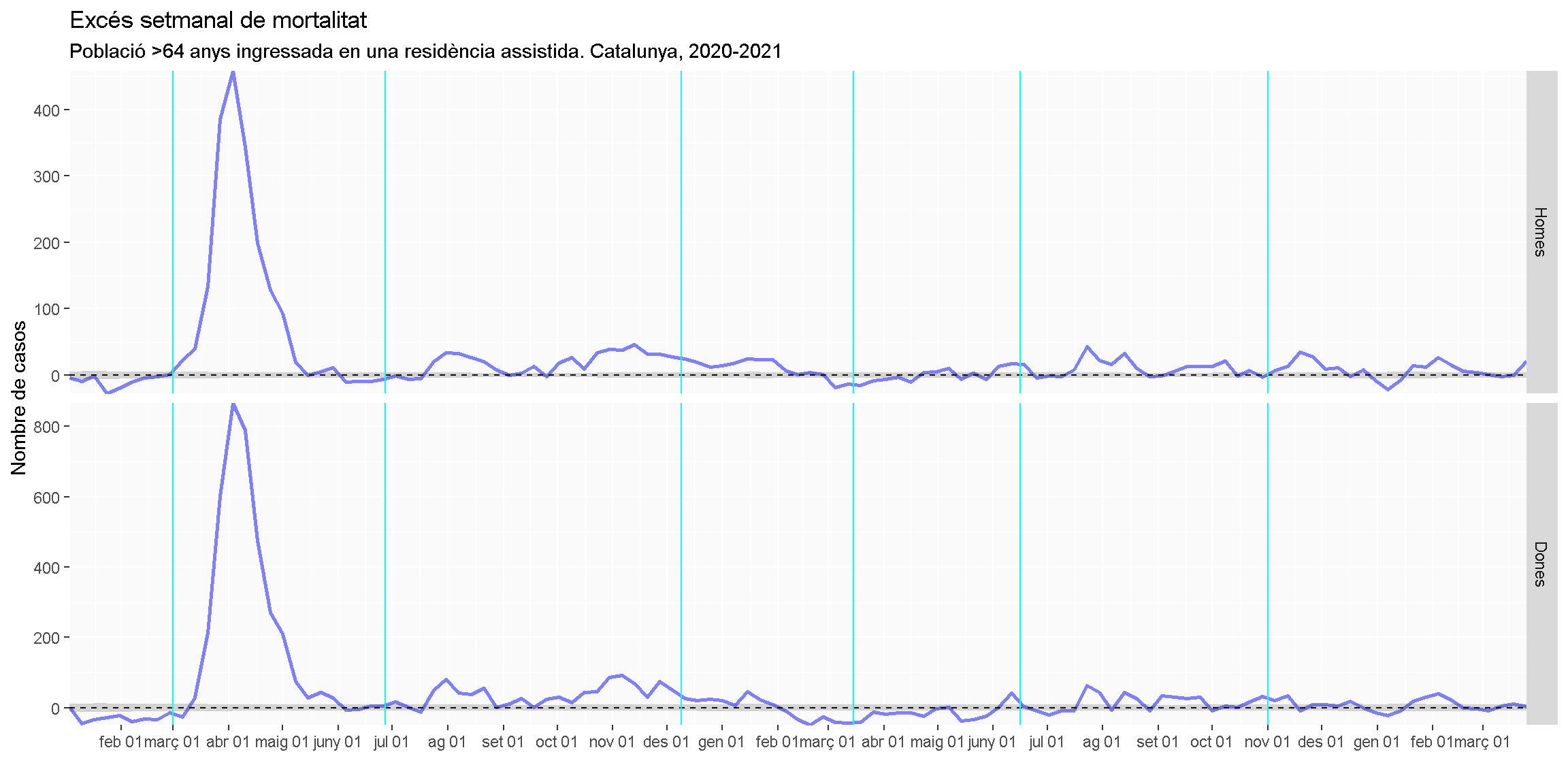
**

## **Figure S4.** Mortality among individuals aged ≥65 years institutionalized in a nursing home during the COVID-19 outbreak, according to age groups.

**A**: Expected and observed weekly mortality rate. **B**: standardized mortality rate (blue line) with the 95% confidence interval (grey area); the dotted line shows the neutrality. **C:** estimated weekly excess deaths; the dotted line shows the zero excess threshold.


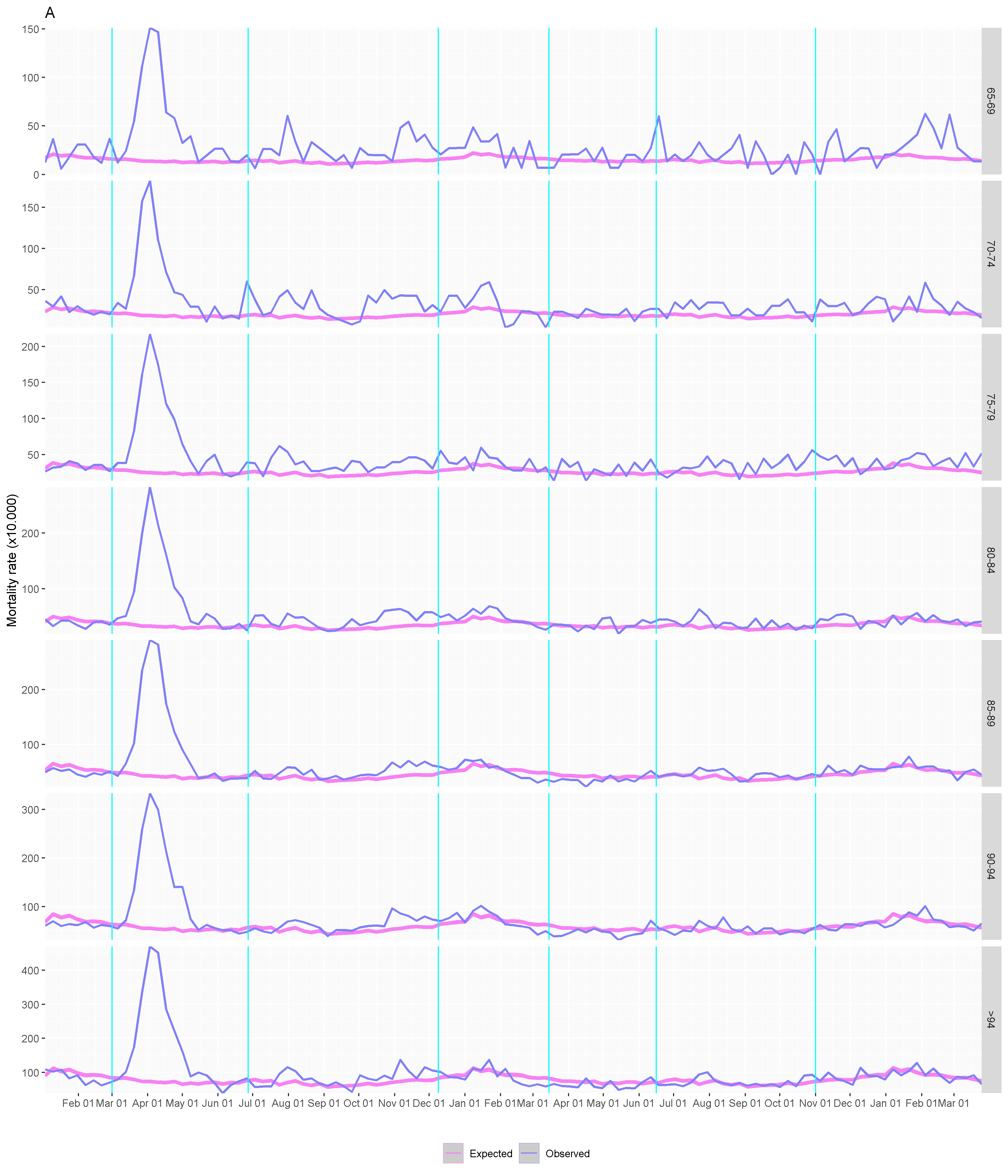

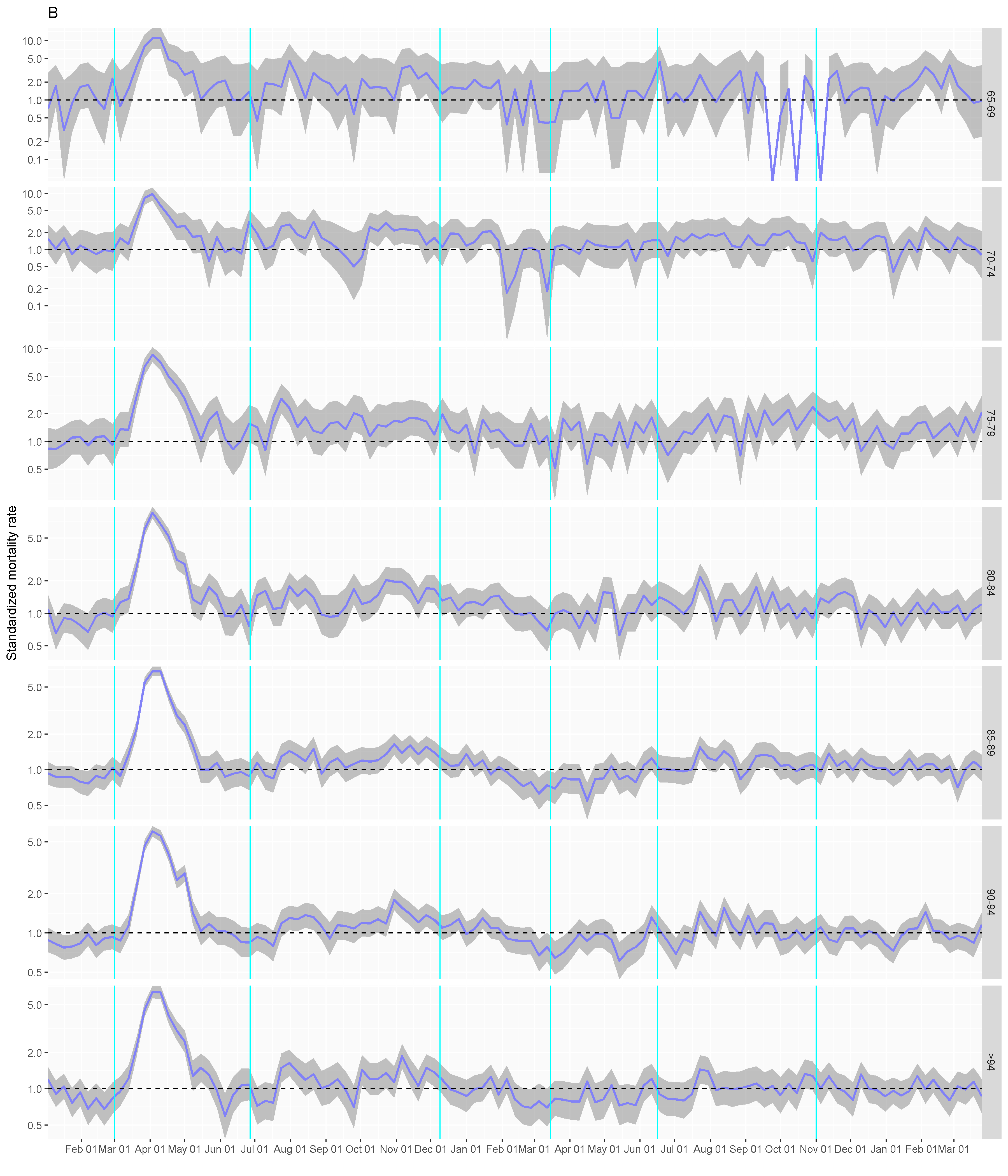

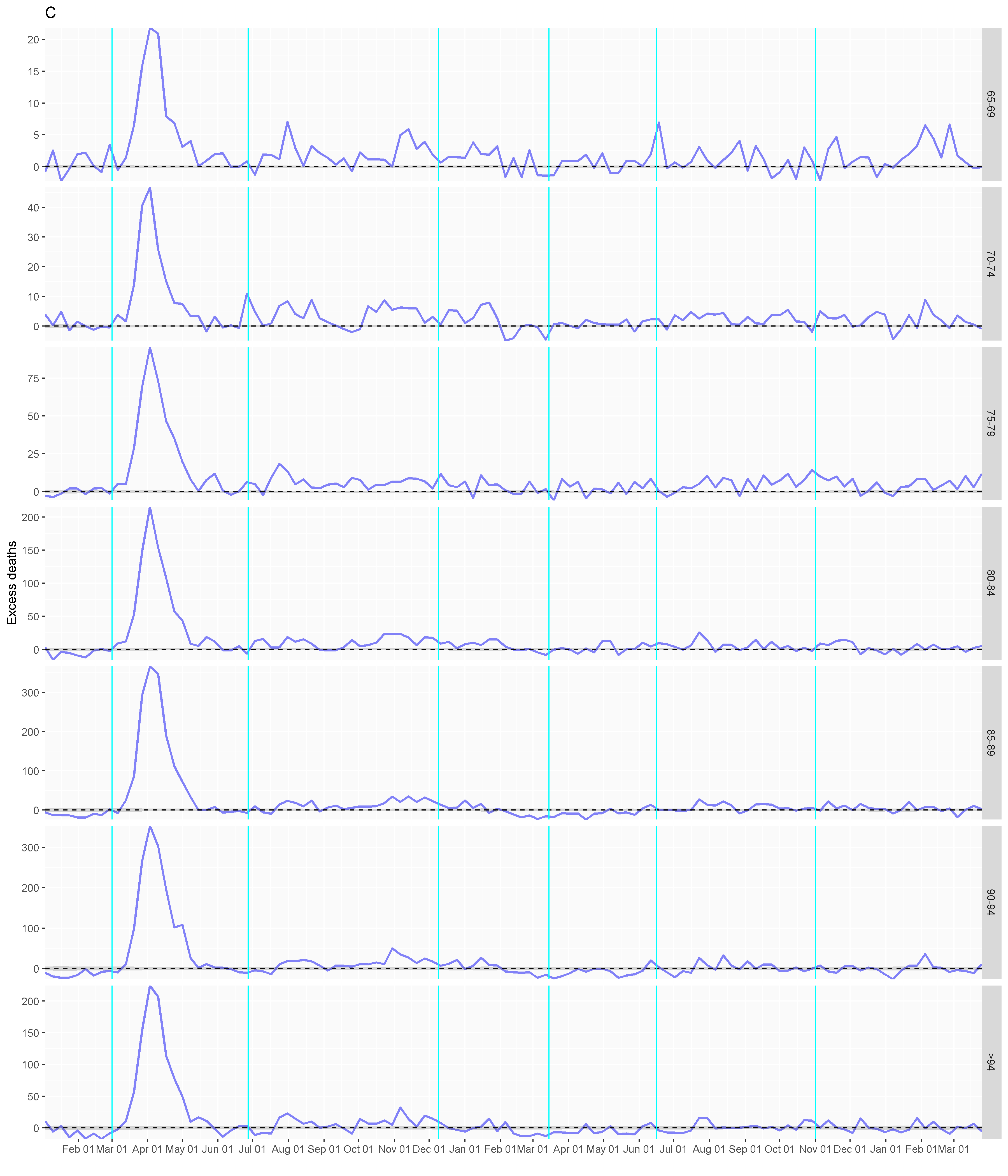


## **Figure S5.** Mortality among individuals aged ≥65 years institutionalized in a nursing home during the COVID-19 outbreak, according to the health risk based on the adjusted morbidity groups.

**A**: Expected and observed weekly mortality rate. **B**: standardized mortality rate (blue line) with the 95% confidence interval (grey area); the dotted line shows the neutrality. **C:** estimated weekly excess deaths; the dotted line shows the zero excess threshold.


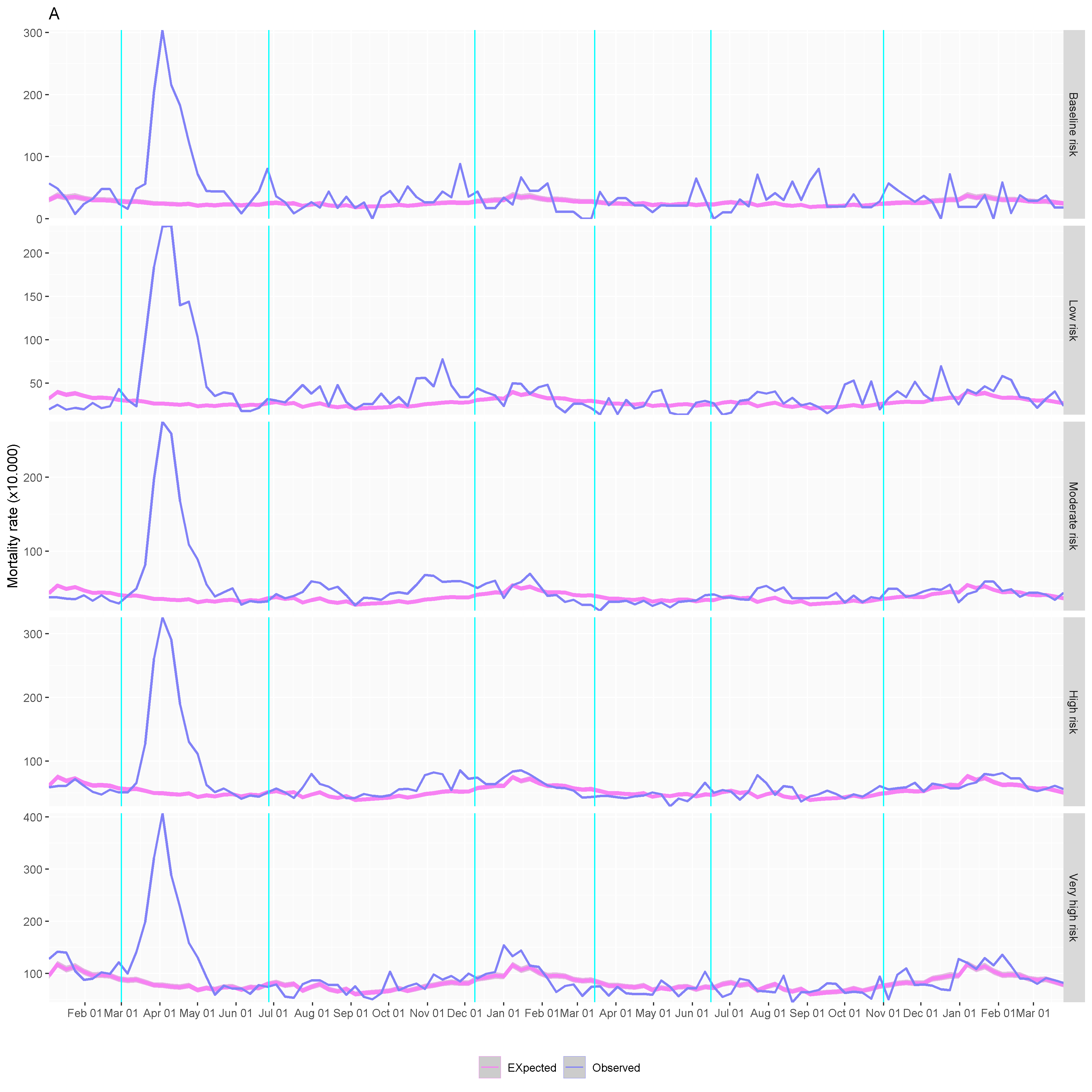

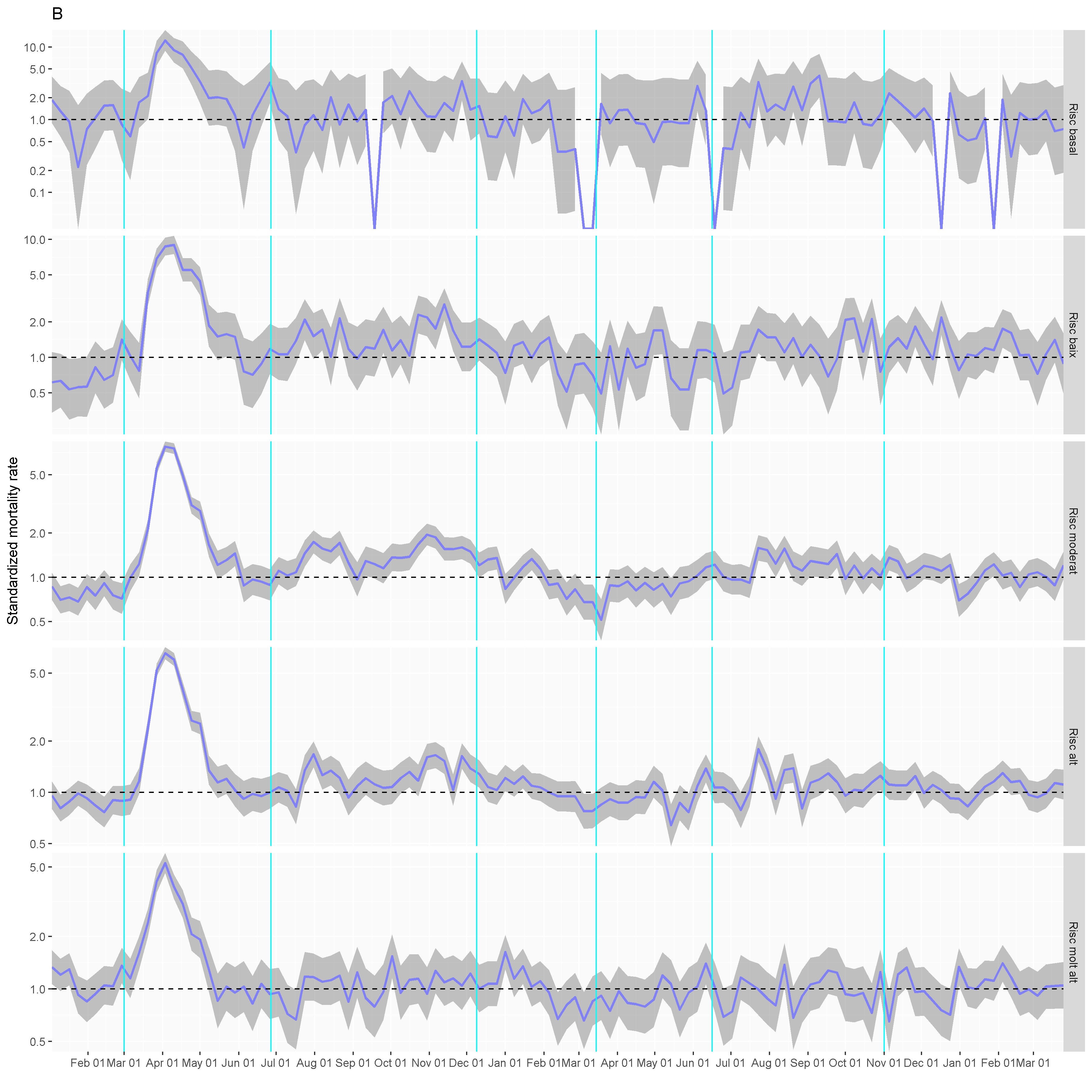

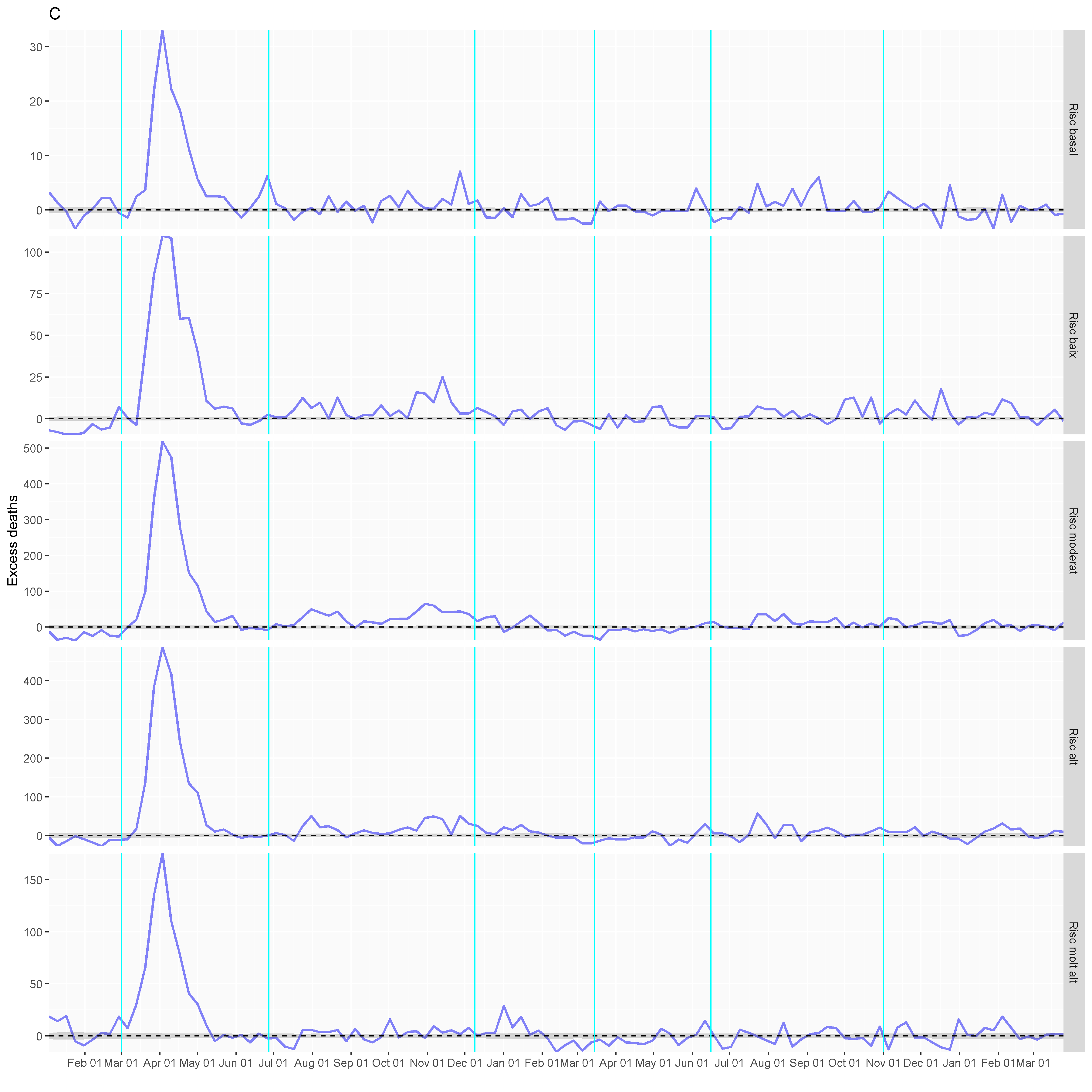

Supplement: Supplementary file 1 [file Data_Sheet_1.DOCX]
